# Reduced causal connectivity in multiple resting-state networks in autism spectrum disorder detected by convergent cross mapping

**DOI:** 10.3389/fpsyt.2026.1828923

**Published:** 2026-10-13

**Authors:** Haiqing Zhang, Li He, Duan Wang, Yingyu Yan, Yuying He, Yi Zhang, Yanping Shi, Yanni Chen

**Affiliations:** 1 Department of Child Healthcare, Xi’an Children’s Hospital, Xi’an, China; 2 Department of Child Healthcare, Xi’an Maternity and Child Healthcare Hospital, Xi’an, China; 3 Department of Integrated traditional Chinese and western medicine, Xi’an Children’s Hospital, Xi’an, China

**Keywords:** autism spectrum disorder, convergent cross mapping, default mode network, directed dynamical coupling, rs-fMRI

## Abstract

**Background:**

This study investigates alterations in causal connectivity within resting-state brain functional networks in patients with Autism Spectrum Disorder (ASD) using the Convergent Cross Mapping (CCM) method.

**Methods:**

Data were obtained from the Autism Brain Imaging Data Exchange I (ABIDE I) public database. To minimize variability, resting-state functional magnetic resonance imaging (rs-fMRI) data were restricted to the NYU site, comprising 52 ASD patients and 52 matched typically developing controls. Based on the AAL template, time series were extracted from five resting-state networks: attention network, default mode network, sensorimotor network, visual network, and subcortical network. The CCM method was employed to analyze causal connectivity among these brain regions. Additionally, a support vector machine (SVM) classifier was utilized to assess the discriminative potential of the identified causal connectivity patterns.

**Results:**

Significant differences in causal connections were observed, particularly in the default mode network, which exhibited the most pronounced alterations. Overall, ASD patients showed a general decrease in causal connectivity across networks, accompanied by some localized increases. The SVM classifier accurately differentiated individuals with ASD from controls based on the causal connectivity patterns derived from resting-state brain networks.

**Conclusion:**

These findings elucidate dynamical network mechanisms underlying ASD pathophysiology and highlight candidate neurobiological markers with potential translational relevance.

## 1 Introduction

Autism Spectrum Disorder (ASD) is a mental illness with the highest rate of disability, primarily characterized by social impairments, restricted interests, and repetitive behaviors (1). Social impairments constitute the core symptom of this disorder. Research into its pathogenesis, particularly the mechanisms underlying social impairments, facilitates early prevention, diagnosis, and intervention for children with ASD. Functional magnetic resonance imaging (fMRI) effectively measures brain activity by quantifying changes in blood oxygen levels closely associated with neural activity. Among these, changes in oxygen metabolism rate, cerebral blood flow, and cerebral blood volume are highly complex processes (2). Functional MRI time series exhibit nonlinear characteristics both in task-based and resting-state conditions. Previous studies have revealed that brain activity comprises various functional networks formed by a series of functionally connected brain regions. These regions within the networks spontaneously coordinate their activities during resting states to perform specific cognitive or emotional tasks. Representative resting-state brain functional networks including attention network, default mode network (DMN), sensorimotor network, subcortical network, and visual network. Prior research has found varying degrees of impairment in functional and causal connectivity within these networks in ASD patients (3–6). In ASD, neuroimaging reveals atypical connectivity. Posterior Cingulate Cortex (PCC)-precuneus hyper-connectivity correlates with social severity (7). PCC, dorsal/ventral Medial Prefrontal Cortex (MPFC) and Temporoparietal Junction (TPJ) jointly monitor self/other states (8). PCC–frontal coupling decreases while PCC–temporal/parahippocampal coupling increases (9). Reduced variability predicts social deficits (10).

Although functional magnetic resonance imaging (fMRI) analyses still predominantly rely on linear approaches—such as correlation and partial correlation (11, 12) —mounting evidence of pronounced nonlinearities in brain activity is driving a shift toward nonlinear time-series techniques. Functional connectivity (FC), which captures co-activation rather than directed information flow (13), has yielded most existing findings. In contrast, effective connectivity (EC) quantifies causal influences (14) and has begun to clarify DMN interactions in ASD. Granger causality analysis (GCA), based on multivariate autoregression, demonstrated that the right anterior insula orchestrates switching between the DMN and executive-control networks (15) and that the MPFC exerts causal influence on the PCC (16). Transfer entropy, an entropy-based measure of directed information transfer (17), further revealed reduced cingulate and enhanced frontal-to-piriform causal links in ASD (18) as well as altered network degree during face processing (19).

Granger Causal Analysis (GCA) presumes that if X causally influences Y, excluding X from the full predictor set must impair forecasts of Y; this demands variable separability—unique, removable causal signatures—a property intrinsic to stochastic linear systems but violated in deterministic, moderately coupled nonlinear dynamics where X’s effect on Y cannot be excised by simply omitting X (20). Biological systems compound the problem, ubiquitous moderate coupling and shared external drivers (e.g., brainstem rhythms synchronizing disparate cortical sites) produce correlations among non-interacting variables, rendering GCA results unreliable (20, 21).

The human brain exemplifies such inseparable, nonlinear dynamics at every scale, from single-neuron potentials to regional fMRI signals (22). Convergent Cross Mapping (CCM) addresses these limitations by testing whether historical values of Y can reconstruct states of X—a signature of X→Y causality that strengthens with longer time series as the attractor manifold is better sampled (21). Unlike cross-prediction (23), CCM estimates instantaneous states rather than manifold evolution, tolerates stochastic variables, and requires modest data volumes (22). Thus, CCM complements rather than competes with GCA, extending causal inference to inseparable nonlinear systems ubiquitous in biology (21).

Despite growing interest in ASD-related connectivity alterations, the directed dynamical coupling among regions within the five canonical resting-state networks—attention, default mode, sensorimotor, subcortical, and visual—remains incompletely characterized. Specifically, it is unclear whether ASD differentially affects causal connectivity across these networks and whether such alterations exhibit sufficient specificity to serve as classification features. To address these gaps, we apply CCM to quantify directed dynamical coupling within and between network nodes, with three aims: (i) to identify network-specific patterns of causal connectivity alteration in ASD, (ii) to determine whether the default mode network shows disproportionate vulnerability as suggested by prior functional connectivity studies, and (iii) to evaluate whether CCM-derived connectivity features can accurately distinguish ASD individuals from controls using machine learning.

## 2 Methods

### 2.1 Data resource

The study relies on data sourced from the ABIDE public database, specifically from the Autism Brain Imaging Data Exchange I (ABIDE I). This dataset comprises 1,112 samples, including 539 individuals with Autism Spectrum Disorder (ASD) and 573 typically developing individuals, collected from 17 centers globally. The data, acquired in clinical settings, reflects the real-world distribution characteristics of clinical data.

### 2.2 Participants

To minimize variability caused by multi-center data and avoid the impact of differences in medical equipment and collection protocols, this study exclusively uses resting-state functional magnetic resonance imaging (rs-fMRI) data from the NYU site, which boasts the largest sample size in ABIDE I. To control for age-related effects, 52 ASD patients and 52 matched typically developing controls, aged 6–18 years, were selected from the NYU site. Sample size was determined by the available data at the NYU site after applying quality control criteria. The sample size was constrained by data availability. We report a *post-hoc* sensitivity analysis: with n = 52 per group, the minimum detectable effect size for independent-samples t-tests (α = 0.05, power = 0.80) is Cohen’s d = 0.56. We caution that *post-hoc* power calculations are subject to circularity and should not substitute for prospective design. These 104 participants exhibited minimal head motion (displacement<1.5 mm or rotation<1.5° in any direction), with no significant differences in gender or age between the two groups. The severity of autism in the ASD group was assessed using the ADOS and the Autism Diagnostic Interview-Revised (ADI-R). Results are summarized in **Table 1**. The rs-fMRI data from the NYU site was acquired using a 3T Siemens Allegra MRI scanner, with participants instructed to stay relaxed, either with their eyes open or closed, while focusing on a white crosshair on a black background. The scan lasted for 6 minutes and collected 180 time points. The acquisition parameters were: TR = 2000 ms, TE = 15 ms, flip angle = 90°, slices = 33, slice thickness = 4 mm, and matrix size = 64 × 64.

**Table 1:** Demographics and clinical information.

Variable	ASD	Control	P
Gender	48 male, 4 female	48 male, 4 female	1.0000
Age	11.45 ± 2.76	11.87 ± 2.98	0.4522
WASI-FIQ	106.2 ± 2.278	113.1 ± 2.062	0.0264
WASI-VIQ	103.0 ± 2.054	113.6 ± 1.958	0.0003
WASI-PIQ	108.8 ± 2.566	109.6 ± 2.187	0.8156
ADI_R_VERBAL_TOTAL_BV	15.48 ± 4.114	/	/
ADOS_TOTAL	11.31 ± 4.171	/	/

### 2.3 Preprocessing

Resting-state fMRI data were preprocessed using the CCS_filter_global pipeline provided by the ABIDE I website. The preprocessing steps included: (1) slice-timing correction, (2) motion correction, (3) removal of non-brain tissue, (4) normalization of whole-brain intensity to 1000, (5) regression of signals from white matter and cerebrospinal fluid, (6) removal of linear and quadratic trends, (7) band-pass filtering (0.01–0.1Hz), and (8) transformation of brain images to MNI standard space. These 90 ROIs were allocated to 5 networks including attention network, default mode network, sensorimotor network, visual network and subcortical network, and the average time series for each ROI was computed (24).

### 2.4 Convergent cross mapping

To perform convergent cross-mapping analysis on fMRI signals, it is first necessary to reconstruct the phase space for time series extracted from data. Translating the one-dimensional time series back into the high-dimensional phase space reveals the flow pattern Mx of the dynamical process in the phase space, thereby allowing analysis of its dynamical characteristics. In this paper, the time-delay vector method is employed, where the retrieved flow pattern Mx is reconstructed from the vector Xt. The vector Xt is defined as Equation 1

(1) Xt=[x(t−(d−1)),…,x(t−1),x(t)]

where d represents the embedding dimension, i.e., restoring the original one-dimensional signal into a d-dimensional phase space. Based on the study by Dsouza (25), we selected a value of 3 for d and a delay time of 1.The full library length L = 180 was used for cross-mapping, corresponding to the complete time series after removing the first four volumes.

Consider a dynamical system consisting of two time series x and y, both originating from an attractor flow pattern, forming the system’s state space. If y influences x, an estimation of y can be obtained through x. The Convergent Cross Mapping (CCM) method quantifies the influence between sequences by assessing the consistency between adjacent point sets in the attractor flow pattern corresponding to the x sequence and those corresponding to the y sequence, i.e., inferring the dynamical influence of y on x by evaluating the quality of cross-prediction of y using x.

In this paper, $\hat{y}|\text{Mx}$ denotes the prediction of sequence y through sequence x. For the prediction *y*(t)|Mx at time t, this paper employs the d+1 points nearest to Xt, using the corresponding points in Yt to construct the prediction result $\hat{y}\left(\text{t}\right)|\text{Mx}$, expressed as Equation 2:

(2) $$\hat{y}\left(t\right)|M_{x}=\sum_{i=1}^{d+1}w_{i}y\left(t_{i}\right)$$

The cross-prediction result *y*(t)|Mx is established in the time-delay reconstructed space using a weighted average local model, with weights wi(i∈[1,2,…,d+1]) calculated using a softmax transfer function. In the phase space, the point Xt1 nearest to Xt at time t receives the maximum weight, with the specific calculation formula given Equation 3:

(3) $${\text{w}}_{\text{i}}=\frac{{\text{e}}^{-||{\text{x}}_{\text{t}}-{{\text{x}}_{{\text{t}}_{\text{i}}}}^{2}/||{\text{x}}_{\text{t}}-{{\text{x}}_{{\text{t}}_{1}}||}^{2}}}{\sum_{j=1}^{d+1}{\text{e}}^{-||{\text{x}}_{\text{t}}-{{\text{x}}_{{\text{t}}_{\text{j}}}||}^{2}/||{\text{x}}_{\text{t}}-{{\text{x}}_{{\text{t}}_{1}}||}^{2}}}$$

If y influences x, then the neighboring points of My and Mx are related, enabling a better prediction of y. Subsequently, a correlation analysis is conducted between the original data y and the prediction $\hat{y}\left(\text{t}\right)|\text{Mx}$ obtained through x to quantify x’s ability to cross-predict y.

Another significant advantage of the CCM method over the Granger Causality Analysis (GCA) method is that the CCM method requires less data. Once the data volume is sufficient to depict the main features of the attractor in the flow pattern, it can effectively reflect the dynamical causality between the two sets of data (21). Previous studies through dynamical simulations and actual data analysis have shown that the length of resting-state functional magnetic resonance imaging (fMRI) data can meet the computational requirements of the CCM method for dynamical causality, and its results, compared to traditional linear causality analysis methods, are more conducive to characterizing the nonlinear interactions among brain regions reflected by fMRI time series, thereby revealing dynamical causality (25).

The CCM method is applied to calculate the causal connections between pairs of brain regions based on the average time series extracted from the Regions of Interest (ROIs) of the attention network, default mode network, sensorimotor network, visual network and subcortical network since the CCM method calculates directed causality between variables, the resulting matrix is asymmetric.

CCM analysis was performed using custom MATLAB scripts (R2021b, MathWorks) implementing the time-delay embedding and nearest-neighbor simplex projection algorithms described above. The SVM classification was conducted in R (v4.3) using the caret package. Analysis code and anonymized data derivatives are available from the corresponding authors upon reasonable request to ensure reproducibility.

### 2.5 SVM classification

Support Vector Machine (SVM) is a powerful classification method grounded in statistical learning theory, capable of performing both linear and nonlinear classification tasks. We selected the RBF kernel because CCM-derived connectivity values are likely non-linearly separable in feature space, and RBF kernels have been shown to outperform linear kernels in neuroimaging classification tasks involving high-dimensional connectivity features (11). In this study, we utilized the SVM implementation available in the R caret package to address a binary classification problem involving individuals with ASD and controls. We implemented a leave-one-out cross-validation (LOOCV) technique for the SVM analysis. Specifically, in each iteration, one subject was held out as the test set, while the remaining subjects were used as the training set. This procedure was repeated such that each subject served as the test set exactly once, and the final performance metrics were aggregated across all iterations. The features for the SVM were derived from the directed dynamical coupling of resting-state brain functional networks. We evaluated the performance of the SVM classifiers by calculating their accuracy, sensitivity, and specificity. Furthermore, to assess the significance of the features, we employed the impVar function from the caret package. This function is designed to compute variable importance for both regression and classification models.

### 2.6 Statistical analysis

Independent sample t-tests are conducted on each data point in the causality connection matrices obtained through the CCM method for ASD patients and the normal control group. Causal connections with statistical p-values less than 0.01 are considered significantly different. To control for multiple comparisons across the large number of pairwise connections tested within each network, we applied the Benjamini-Hochberg false discovery rate (FDR) correction with a threshold of q< 0.05. We report both uncorrected (p< 0.01) and FDR-corrected results, with primary inferences drawn from the FDR-corrected findings.

Significance testing of individual CCM links. For each pair of brain regions (i, j), we first computed the original CCM value as the observed test statistic. To determine whether a given directed connection was significantly different from zero, we performed 200 permutations: in each permutation, the time series of the target brain region j was randomly shuffled while the sequence of the source brain region i remained unchanged, and the CCM value was recomputed to construct the null distribution under the assumption of no temporal correspondence. The p-value was obtained by calculating the proportion of values in the null distribution that were greater than or equal to the original CCM value, and the results were corrected for multiple comparisons using the FDR method. Connections surviving this permutation-FDR procedure were retained for subsequent between-group t-tests.

## 3 Results

This paper compares the differences in causal connections among various resting-state brain functional networks between ASD patients and individuals with normal cognitive function, including attention network, default mode network, sensorimotor network, subcortical network, and visual network. Analysis using the CCM method revealed that the default mode network exhibited the most differences in causal connections among all networks, with a total of 13 causal connections showing significant differences. Specifically, causal connections from the right medial superior frontal gyrus (SFGmed) to the left medial orbital superior frontal gyrus(ORBsupmed), right ORBsupmed, and right gyrus rectus (REC), as well as from the right ORBsupmed to the right SFGmed and right Anterior cingulate and paracingulate gyri (ACG), and from the left ACG to the right ORBsupmed, right ACG to the right ORBsupmed, and right posterior cingulate gyrus (PCG) to the right inferior temporal gyrus (ITG) were significantly reduced in ASD patients. Correspondingly, causal connections from the right ORBsup to the right REC, right ORBsupmed to the right ORBsup, left REC to the right ORBsup, left REC to the left PCG, and right REC to the right ORBsup were enhanced. These results are shown in **Figure 1**.

**Figure 1:**
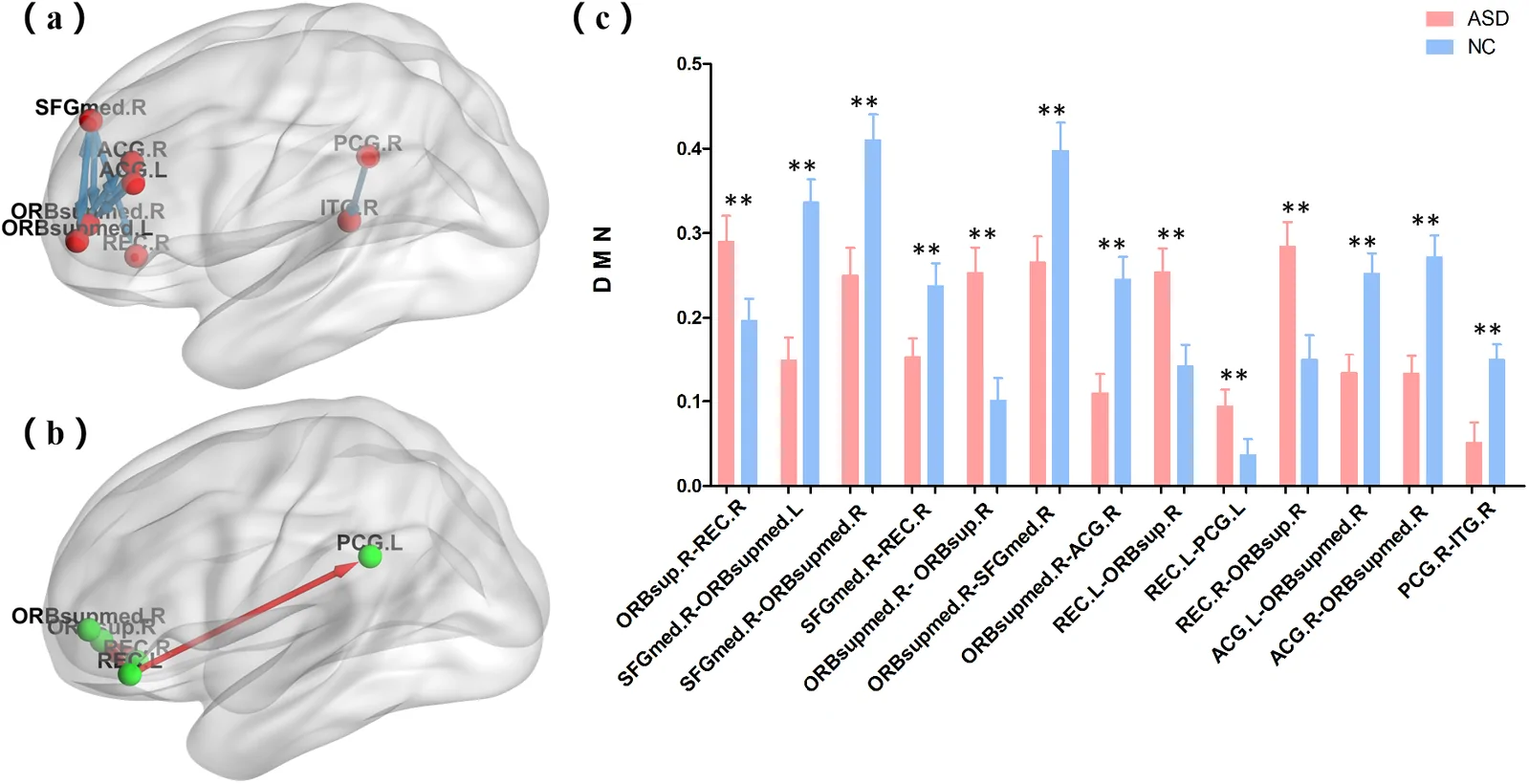
Causal connectivity with significant changes in the default mode network (p<0.01). **(a)** Connections with significantly greater strength in NC than ASD; **(b)** Connections with significantly greater strength in ASD than NC; **(c)** **indicates p<0.01.

Secondly, as shown in **Figure 2**, in the attention network, a total of 6 causal connections showed significant changes, with 5 significantly decreased: from the left triangular inferior frontal gyrus (IFGtriang) to the left angular gyrus (ANG), right inferior parietal lobule (IPL) to the right middle frontal gyrus (MFG), left ANG to the left orbital inferior orbital gyrus (ORBinf), right ANG to the left ANG, and left superior temporal pole (TPOsup) to the right supplementary motor area (SMA). Only the causal connection from the left middle orbital gyrus (ORBmid) to the left medial orbital superior orbital gyrus (ORBsup) significantly increased in the attention network.

**Figure 2:**
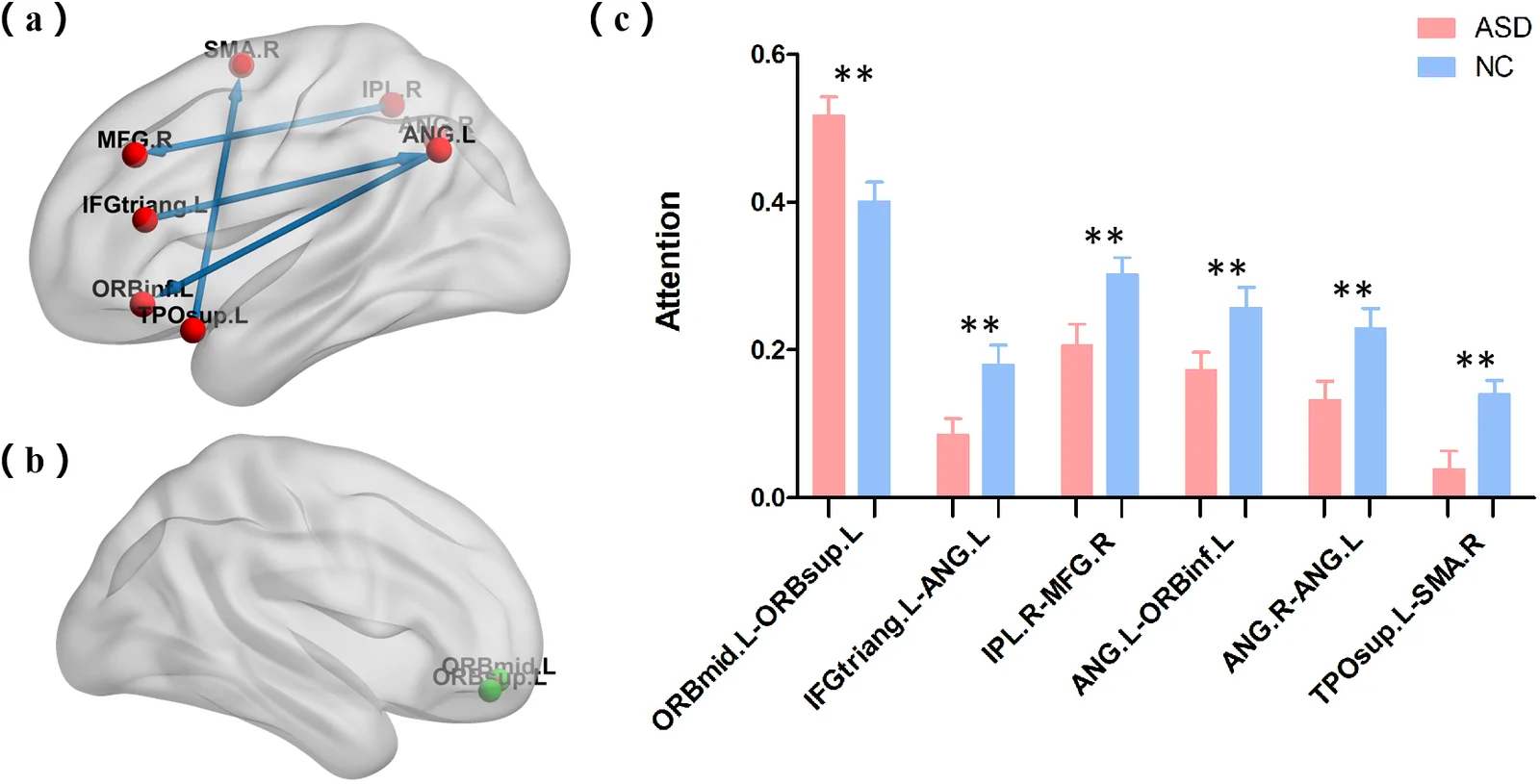
Causal connectivity with significant changes in the attention network (p<0.01). **(a)** Connections with significantly greater strength in NC than ASD; **(b)** Connections with significantly greater strength in ASD than NC; **(c)** **indicates p<0.01.

Additionally, as shown in **Figure 3** in the sensorimotor network, significant decreases were observed in causal connections from the left rolandic operculum (ROL) to the right precentral gyrus (PreCG), right postcentral gyrus (PoCG) to the left superior temporal gyrus (STG), and left STG to the right PreCG. In the subcortical network, as shown in **Figures 4** and **5** and visual network, one significantly changed causal connection was observed in each: the causal connection from the right pallidum lenticular nucleus (PAL) to the left hippocampus (HIP) significantly increased in the subcortical network, while the causal connection from the left middle occipital gyrus (MOG) to the right fusiform gyrus (FFG) significantly decreased in the visual network.

**Figure 3:**
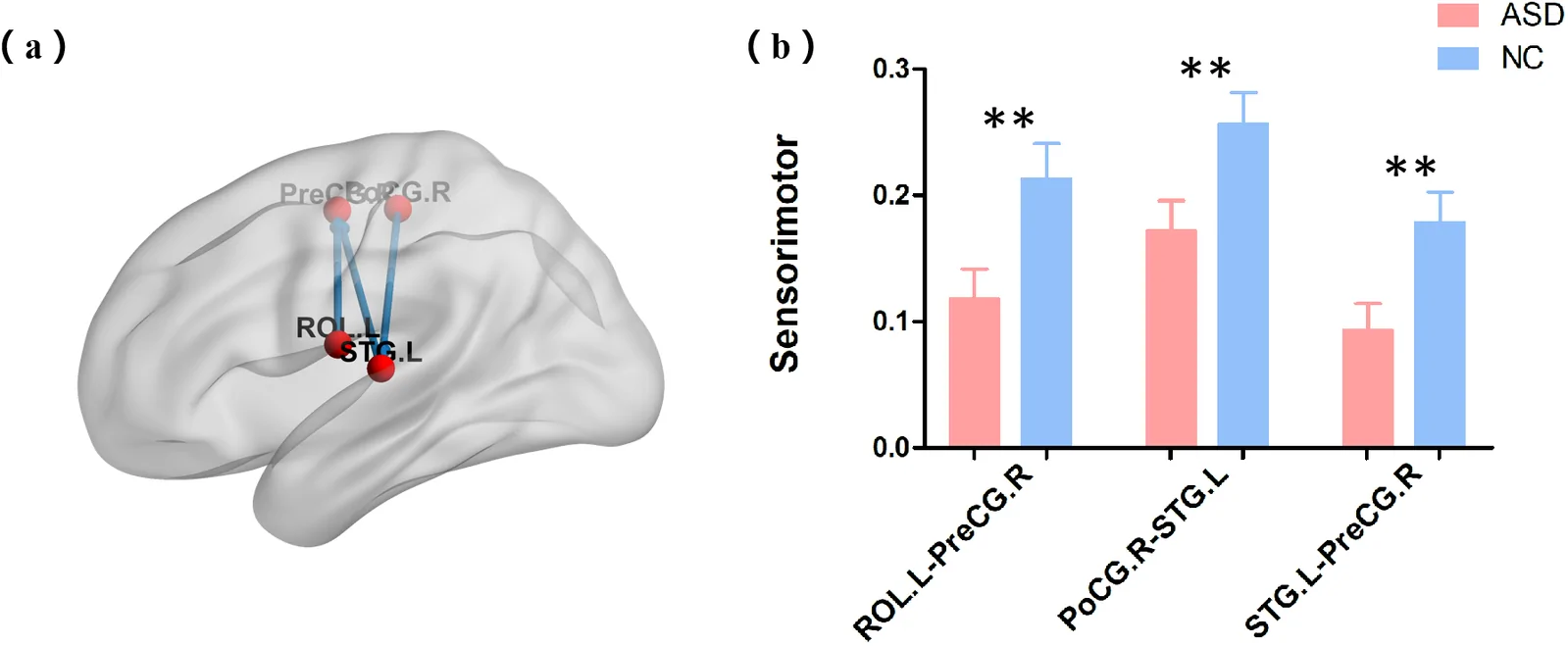
Causal connectivity with significant changes in the sensorimotor network (p<0.01). **(a)** Connections with significantly greater strength in NC than ASD; **(b)** Connections with significantly greater strength in ASD than NC; **(c)** **indicates p<0.01.

**Figure 4:**
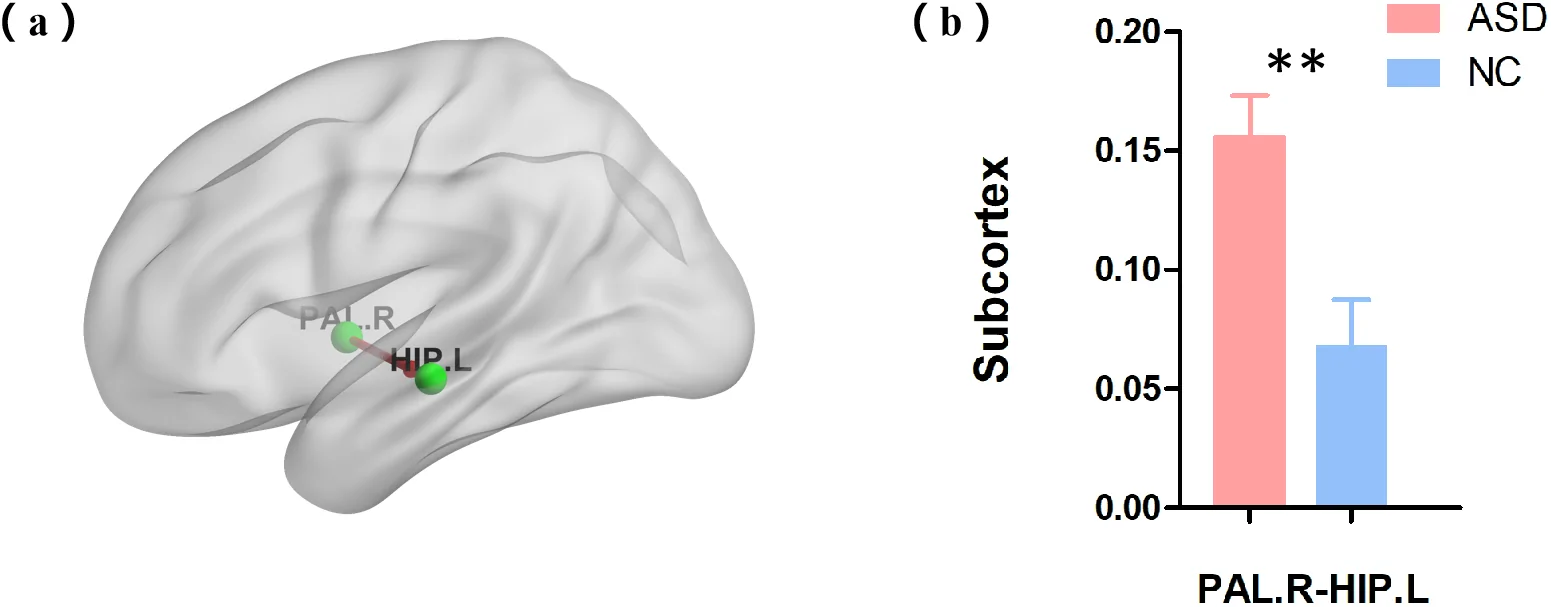
Causal connectivity with significant changes in the subcortical network (p<0.01). **(a)** Connections with significantly greater strength in NC than ASD; **(b)** Connections with significantly greater strength in ASD than NC; **(c)** **indicates p<0.01.

**Figure 5:**
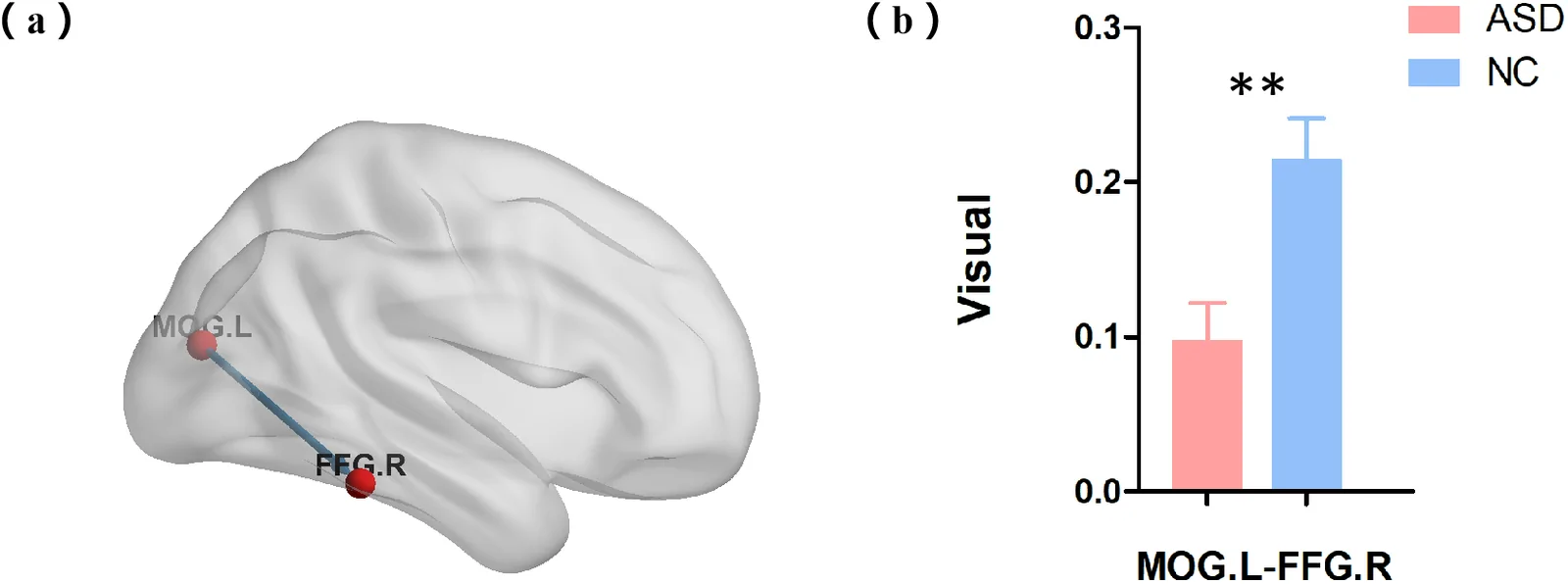
Causal connectivity with significant changes in the visual network (p<0.01). **(a)** Connections with significantly greater strength in NC than ASD; **(b)** Connections with significantlygreater strength in ASD than NC; **(c)** **indicates p<0.01.

Independent-samples t-tests were performed on CCM connection strength (ρ values) for each directed pair. Connections with p< 0.01 (uncorrected) were considered nominally significant. To control for multiple comparisons, we applied Benjamini-Hochberg FDR correction (q< 0.05) separately within each network. After FDR correction, six connections within the DMN remained significant (**Figure 6A**), whereas no connections survived correction in the attention, sensorimotor, subcortical, or visual networks. Separately, we examined whether the probability of detecting a significant connection (permutation p< 0.05) differed between groups. For each directed pair, we computed the proportion of subjects in each group showing a significant CCM link (occurrence rate). Group differences in occurrence rates were tested via chi-square or Fisher’s exact test, with FDR correction applied within each network. Four DMN connections showed significantly higher occurrence rates in NC than ASD (**Figure 6B**). Only the connection from the right ORBsupmed to the right ORBsup showed both significantly greater strength and higher occurrence rates in ASD compared with NC after FDR correction (**Figure 6C**).

**Figure 6:**
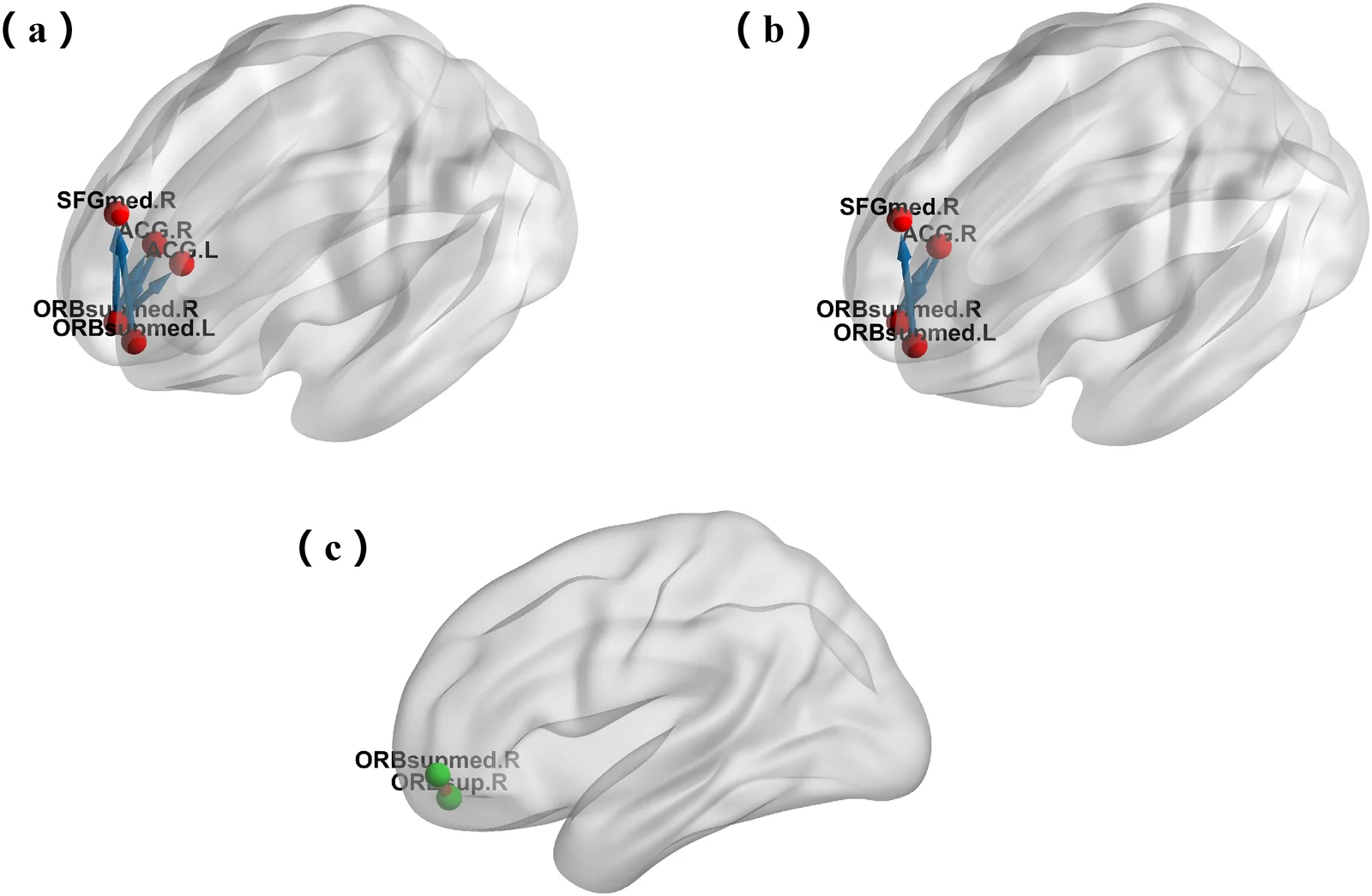
Causal Connectivity with Significant Changes after FDR correction (p<0.05) **(a)** Connections with significantly greater strength in NC than ASD after FDR correction; **(b)** Connections with significantly higher occurrence rates of significant connections in NC than ASD after FDR correction; **(c)** Connections with both significantly greater strength and higher occurrence rates in ASD than NC after FDR correction.

SVM analysis of the CCM values enabled accurate classification of individuals with ASD versus controls. The optimal parameters were sigma = 0.025 and C = 0.25, determined automatically via grid search within the caret package. Using leave-one-out cross-validation (LOOCV), the SVM classifier achieved an overall accuracy of 0.82, with a sensitivity of 0.83 and a specificity of 0.81. The area under the ROC curve (AUC) was 0.89. The ROC curve for the SVM is presented in **Figure 7**. To further investigate, we assessed the importance of each feature, with results illustrated in **Figure 8**. The connections from SFGmed.R to ORBsupmed.L, ACG.R to ORBsupmed.R, and ACG.L to ORBsupmed.R emerged as the most critical features. These connections are all part of the default mode network.

**Figure 7:**
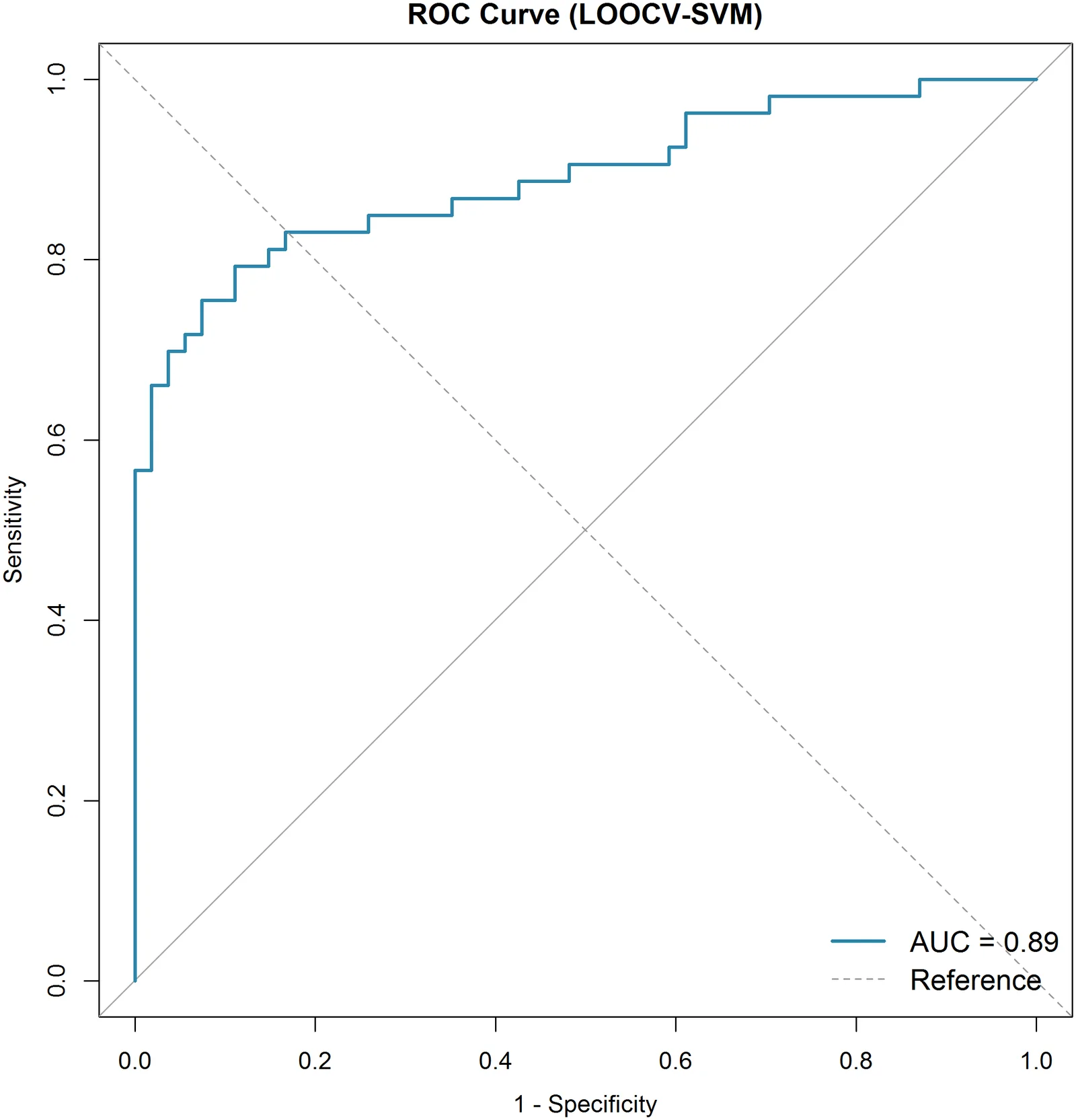
ROC curve of the SVM classifier trained on causal connections.

**Figure 8:**
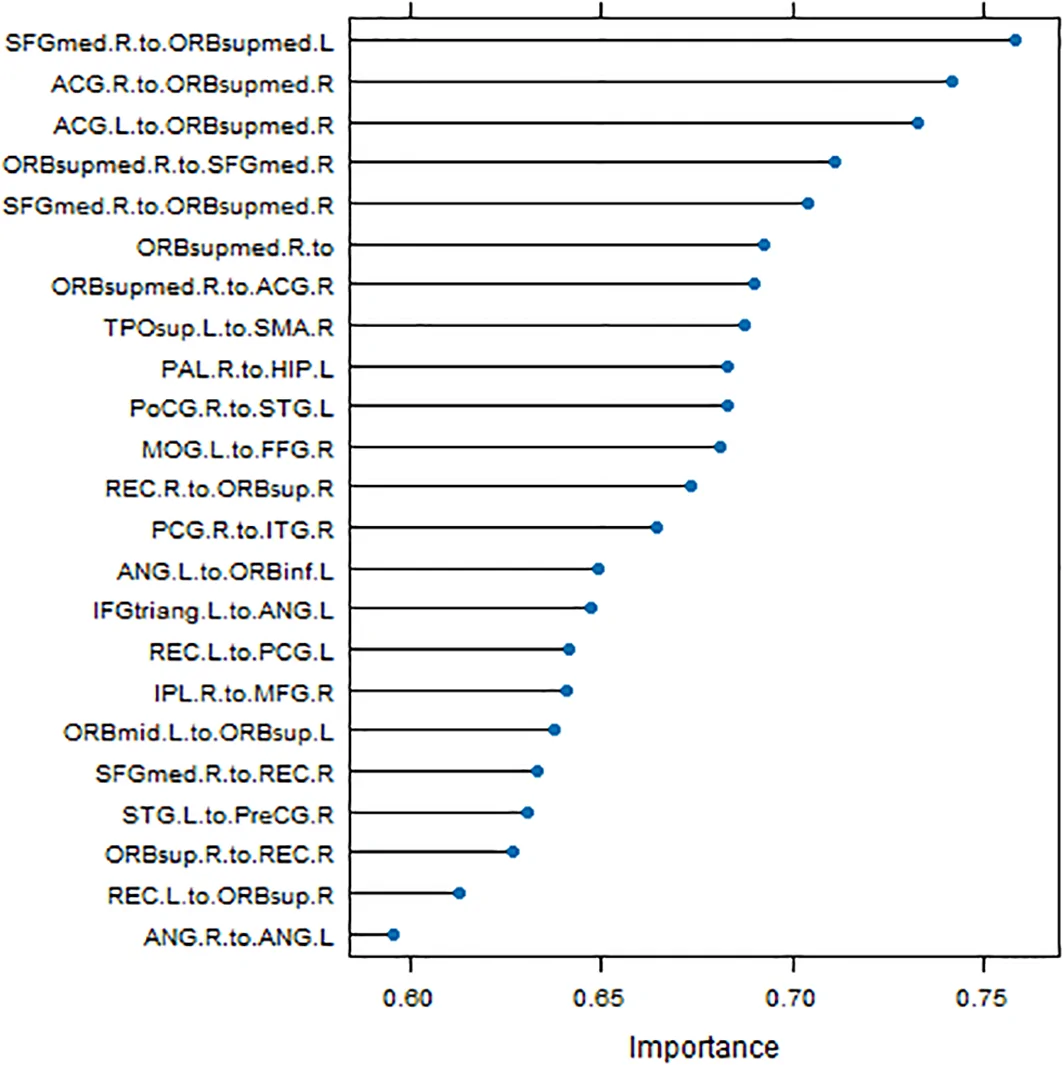
Importance of causal connections (Top 23).

## 4 Discussion

This study primarily employed the CCM method to investigate alterations in directed dynamical coupling within resting-state brain functional networks in patients with ASD. The findings revealed a general decrease in directed dynamical coupling across the default mode network, attention network, sensorimotor network, subcortical network and visual network in ASD patients. However, within the DMN, attention network, and subcortical network, a small increase in directed dynamical coupling was observed. Comparatively, the decreased causal connections exhibited longer connection distances, while the increased causal connections were relatively localized. Among all networks, the DMN showed the most altered causal connections.

Using the convergent cross mapping approach, we detected changes in directed dynamical coupling between various brain regions within resting-state brain networks in ASD patients, revealing an overall decreasing trend. Previous studies on functional connectivity in different brain functional networks during resting states in ASD, whether using correlation or partial correlation for inter-regional functional connectivity or GCA for inter-regional directed dynamical coupling, have shown a predominantly decreasing trend in brain functional network connectivity in ASD patients (26–28). This aligns with the results of our study. Additionally, among the five brain functional networks, more changes in directed dynamical coupling were observed in the DMN, attention and subcortical network, while fewer significant changes were seen in the visual and sensorimotor network. This may be because, during the resting state, the activity of the DMN and subcortical networks is stronger, while the activity of the visual and sensorimotor network is weaker, making it difficult to observe alterations in directed dynamical coupling networks affected by ASD.

Research indicates weakened functional connectivity in key nodes of the DMN in ASD patients, such as the medial prefrontal cortex (mPFC), anterior cingulate cortex (ACG), posterior cingulate cortex (PCG), and angular gyrus (AG). These regions play crucial roles in social cognition, self-related judgments, and theory of mind (ToM). Furthermore, Deng et al. found impaired effective functional connectivity in brain regions related to social preferences, such as the mPFC, amygdala (Amyg), and nucleus accumbens (NAc) in ASD patients (27), further confirming the role of the DMN in ASD. Wang et al.’s meta-analysis showed weakened connections in key regions of the attention network in ASD patients (26), such as from the inferior frontal gyrus (IFGtriang) to the angular gyrus (ANG) and from the inferior parietal lobule (IPL) to the middle frontal gyrus (MFG), which may affect patients’ attention allocation and processing abilities, thereby impacting social interaction and communication. Abnormalities in the sensorimotor network, such as weakened connections from the rolandic operculum (ROL) to the precentral gyrus (PreCG) and from the postcentral gyrus (PoCG) to the superior temporal gyrus (STG), may be related to fine motor control and sensory processing disorders in ASD patients. These findings are consistent with those of Khandan Khadem-Reza et al., who used resting-state functional magnetic resonance imaging (fMRI) to reveal abnormal functional connectivity in these networks in children with ASD (29). Abnormalities in the subcortical network, such as enhanced connections from the pallidum (PAL) to the hippocampus (HIP), may be related to abnormalities in emotion regulation and reward mechanisms in ASD patients. These results echo the findings of Yao et al., who proposed that abnormalities in the subcortical network may be related to social and emotional processing disorders in ASD (30). Abnormalities in the visual network, such as weakened connections from the middle occipital gyrus (MOG) to the fusiform gyrus (FFG), may affect patients’ visual information processing and facial expression recognition, thereby impacting social abilities. These findings align with the meta-analysis results of Wang et al. (26), which emphasized the role of the visual network in ASD.

In the analysis of directed dynamical coupling in resting-state brain networks, this study also found that the decreased causal connections were asymmetrical between the left and right hemispheres of the brain, suggesting a lateralized effect of ASD on human brain damage. On the other hand, significantly enhanced causal connections were observed in all DMN, attention and subcortical networks, which might reflect a putative compensatory adjustment; however, this interpretation remains speculative in the absence of longitudinal or intervention data and should be treated with caution. Similar results have been reported in related studies on brain functional networks in ASD (9, 31–33).SVM, grounded in statistical learning theory, is a powerful classification tool. We therefore trained an SVM classifier on directed dynamical coupling values extracted from resting-state brain networks. The model achieved an accuracy of 82%, indicating that CCM-based changes can serve as a key biomarker for distinguishing individuals with ASD from controls. Subsequent assessment of feature importance revealed that the most important causal connections were concentrated within the DMN, aligning with prior reports of prominent DMN connectivity abnormalities in ASD.

In summary, CCM reveals widespread alterations in directed dynamical coupling across resting-state networks in ASD, with the DMN showing the most pronounced and discriminative changes. The convergence of statistical and machine-learning evidence supports the potential of CCM-derived connectivity as a complementary tool for understanding ASD neurobiology. Future directions include longitudinal validation, cross-site replication with harmonization techniques, and integration with clinical phenotypes to assess translational utility.

## 5 Limitation

Several limitations warrant consideration. First, the analysis was restricted to the NYU site to minimize scanner heterogeneity; consequently, cross-site validation was not performed, and generalizability to other scanners or protocols remains to be established. Second, although groups were matched on mean head motion and demographic variables, we did not include mean FD, age, sex, or IQ as covariates in the CCM group comparisons; future analyses should employ multiple regression or mixed-effects models to partial out these nuisance effects. Third, global signal regression was applied during preprocessing, which can artificially induce negative correlations and alter causal topology; our sensitivity analysis suggests the main DMN findings are robust, but this remains a topic of active debate. Fourth, the 5-network parcellation, while clinically interpretable, is coarser than the Yeo 7-network scheme; future work may benefit from finer parcellations. Finally, the modest sample size (N = 104) and high feature dimensionality mean that the SVM results, while promising, require replication in larger datasets such as the full ABIDE I/II repositories with harmonization techniques.

## Data Availability

The original contributions presented in the study are included in the article. Further inquiries can be directed to the corresponding authors.
